# Motivations and barriers for clinical mental health help-seeking in Bangladeshi university students: a cross-sectional study

**DOI:** 10.1017/gmh.2022.24

**Published:** 2022-05-10

**Authors:** Munjireen S. Sifat, Naima Tasnim, Nushrat Hoque, Sandra Saperstein, Richard Q. Shin, Robert Feldman, Kirsten Stoebenau, Kerry M. Green

**Affiliations:** 1 TSET Health Promotion Research Center, University of Oklahoma Health Sciences Center, Oklahoma City, OK; 2 BRAC University, Dhaka, Bangladesh; 3 The Pennsylvania State University, University Park, PA, USA; 4 Department of Behavioral and Community Health, University of Maryland School of Public Health, College Park, MD, USA

**Keywords:** Bangladesh, barriers and access to care, global mental health, mental health, motivation, self-determination theory, university students

## Abstract

**Background:**

University and college students are vulnerable to developing depressive symptoms. People in low-income countries are disproportionately impacted by mental health problems, yet few studies examine routes to accessing clinical services. Examining motivation and barriers toward seeking clinical mental health services in university students in Bangladesh is important.

**Method:**

Using a cross-sectional survey (*n* = 350), we assess the relationship between the constructs of autonomy, relatedness, and competency toward using clinical mental health practices (i.e. using professional resources, taking medication) with (1) positive views, (2) perceived need, and (3) use of clinical mental health services among Bangladeshi university students.

**Results:**

Results showed that the perceived need for mental health support was the predictor of the largest magnitude (aOR = 4.99, *p* = 0.005) for using clinical services. Having a positive view of clinical services was predictive of clinical service use (aOR = 2.87, *p* = 0.033); however, that association became insignificant (*p* = 0.054) when adjusting for the perceived need for mental health care. Of the SDT constructs, social influences were predictive of perceiving a need for mental health support, and mental health knowledge was predictive (aOR = 1.10, *p* = 0.001) of having a positive view of clinical mental health care.

**Conclusion:**

Our findings show that knowledge of mental health is associated with positive views of mental health services, and that higher levels of stress and the presence of people with mental health problems are associated with the perception of a need for mental health care, which is ultimately responsible for using the services.

## Introduction

The global burden of mental health has become an increasingly important topic to understand. Mental health disorders have increased in prevalence globally, across diverse demographics, cultures, and political situations (Patel *et al*., 2018). The burden of mental illness is disproportionately high in low- and middle-income nations. Some low-income country populations, particularly in Asia (Lauber and Rössler, 2007; Giasuddin *et al*., 2015), believe that mental health issues result from religious, familial, or cultural disobedience (Ndetei *et al*., 2011). This belief is associated with stigma around mental health (Uddin *et al*., 2019) leading to decreased use of professional mental health care (Koly *et al*., 2021). Therefore, determining how to promote positive views and utilization of mental health care in low- and middle-income nations is critical to increase mental wellbeing in this population.

Mental disorder rates generally rise in young adulthood. In Bangladesh, early adulthood is the most vulnerable period for depressive symptoms (Arafat, 2019). Given the high levels of stress college students face (Eisenberg *et al*., 2011) and the link between stress and depression (Kessler *et al*., 2007; McGorry, 2011), college students are at high risk of developing depressive symptoms (LeMoult, 2020). Early management of depressive symptoms is essential, as treatments can be less effective as the duration of depression increases (Bukh *et al*., 2013).

Several studies have assessed depression in Bangladeshi university students using the Patient Health Questionnaire (PHQ-9). In a study of 665 university students, Hossain *et al*. (2019) found that 74.1% had mild to severe depression. Koly *et al*. (2021) found that 47% of the sample met the criteria for depression and that poor academic performance and excessive use of social media were the most common factors that were associated with depression in college students. Islam *et al*. (2020) showed that 69.5% of first-year students (*n* = 400) experienced moderate to severe depression. Using Beck's Depressive Inventory, Sayeed *et al*. (2020) discovered, in a cross-sectional study of 404 college students, that 47.5% had depression and 8.7% had attempted suicide. Despite strong data supporting the prevalence of depression among Bangladeshi university students, there is little research on utilization of mental health services to reduce depressive symptoms.

Despite the prevalence of mental health disorders, there has historically been a lack of both health care providers that are trained in psychiatric health (World Health Organization, 2007). A report of the assessment of the mental health system in Bangladesh using the World Health Organization – Assessment Instrument for Mental Health Systems (WHO-AIMS) was undertaken in 2007 to examine the current situation in Bangladesh regarding mental health. Bangladesh has one main mental health hospital, and 50 outpatient mental facilities, but there is no follow-up care provided for patients once they leave the facility (WHO-AIMS). Further, only 4% of medical doctors in Bangladesh are trained in mental health, and 2% of nurses have specific mental health training (World Health Organization, 2007), and only 1–20% of primary care physicians make at least one referral to a mental health professional. However, in recent years, there has been a push to scale up mental health services in low-income countries by global leaders such as the WHO, the Lancet, and the World Bank.

It is important to understand the motivations and hesitancies toward seeking and receiving mental health care, particularly in populations where stigma levels are historically high. A systematic review of published literature related to mental health disorders and mental health services in Bangladesh indicated that there are low community awareness of mental health disorders, negative attitudes toward treatment, and low priority for treatment even among those affected by mental health disorders (Hossain *et al*., 2014). Low awareness of mental health is troubling, because if one does not recognize the need for care, they are less likely to receive care (Bilican, 2013), as such perceived need is an important outcome to examine and is an important first step to receiving care. Positive attitudes toward clinical mental health care are linked to mental health-seeking behavior (Cheng *et al*., 2018), therefore promoting mental health awareness and the use of services is critical, as research shows that mental health services such as therapy and medication are effective (Hollon *et al*., 2002; Hollon *et al*., 2005). For example, one study found that cognitive behavioral therapy significantly improved negative mental health symptoms related to post-traumatic stress, depression, and anxiety when compared to people who did not engage in therapy (Mueser *et al*., 2008). Further, medications such as sertraline have been shown to improve post-traumatic stress disorder symptomology when compared to patients taking a placebo (Davidson *et al*., 2001).

Nonclinical strategies including adaptive coping skills and social support have been found to improve an individual's mental health. Coping skills can improve mental health, particularly under tough situations (Meyer, 2001; Garcia *et al*., 2018). There is minimal research on Bangladeshi university students' coping mechanisms. During the COVID-19 pandemic, one study found that college students with ‘high extraversion, agreeableness, conscientiousness, openness and low neuroticism’ had higher abilities to participate in healthful behavior (Ahmed *et al*., 2021). Understanding present coping strategies is crucial to develop mental health interventions for Bangladeshi students.

## Theory

The Self-Determination Theory (SDT) is a useful framework to understand the motivation to engage in a particular behavior. SDT states that motivation for a behavior derives from three natural and psychological needs: competence, psychological relatedness, and autonomy (Vansteenkiste *et al.*, 2004). The construct of competence refers to the feeling that a person has a sense of mastery in their actions, and can be assessed by examining one's perceived knowledge on the subject. Researchers define the construct of ‘mental health knowledge’ based on participants' knowledge of the symptoms and effects of mental health illnesses that are defined by Western countries, like the United States, but these definitions are not widely established in non-Western countries (Sue *et al*., 2009). Therefore, when studying mental health in Bangladesh, it is critical to focus on the sample's culture and language around mental health (Rodrigo, 2015). Relatedness refers to a sense of belonging to others, such as feeling cared for, or connected, and a sense of mutual concern. Actions are self-initiated and self-endorsed when they are autonomous (Vansteenkiste *et al.*, 2004). Autonomy and intrinsic motivation have both been found to have key influences on mental health (Gagné and Deci, 2014). Self-direction and choice are supposed to fuel motivation (Ryan and Deci, 2000) and self-efficacy (Bandura, 1986). Relating competency, relatedness, and autonomy to the constructs of positive views of clinical services, perceived need for mental health support, and the actual seeking of clinical support can help target messaging for mental health care.

**Fig. 1.**
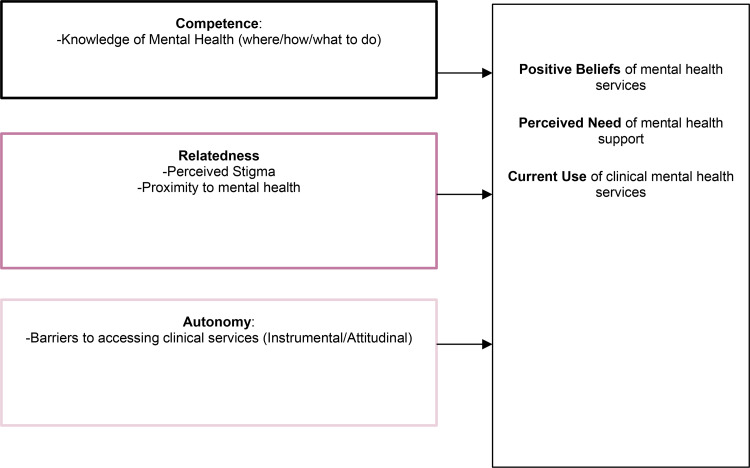
Conceptual model based on self-determination theory.

The perceived need for mental health support is conceptualized as a motivation driven by perceived usefulness of the support; whether one views clinical services positively is conceptualized as a value-driven motivation. Using the components of SDT (see Fig. 1 for the conceptual model utilized), this study reports the prevalence of mental disorders and wellness practices in a sample of Bangladeshi university students and identifies the motivational factors toward self-identification of need for mental health support, positive views of support, and clinical help-seeking.

## Aims and hypotheses

This study's main aims assess the relationship between autonomy, relatedness, and competency toward using clinical mental health practices (i.e. using professional resources, taking medication) with (1) positive views toward clinical mental health services, (2) perceived need of mental health support, and (3) use of clinical mental health services among Bangladeshi university students. It is hypothesized that greater autonomy, greater relatedness, and greater competency are associated with use of clinical services, positive beliefs toward using clinical services, and perceived need for mental health support.

## Methods

### Study sample

An online survey was distributed to current Bangladeshi university students aged 18 or older. Faculty from institutions around Bangladesh emailed students to complete the anonymous survey. Invitations were also posted on university Facebook pages. The poll was open for 2 months, from January to February 2020, and the final dataset included 350 student replies.

### Survey development

Native Bangla speakers were consulted to analyze the initial English survey and prepare a culturally sound Bangla translation. An interview guide for *n* = 5 cognitive interviews with the target demographic was created. Participants were asked how they defined mental health and to explain their interpretation of the survey queries during the cognitive interviews. Items that were difficult to understand or culturally unsuitable were altered and pilot tested (*n* = 10) before final distribution.

## Measures

### Dependent variables

There were three primary outcomes: clinical mental health practices, perceived need for mental health help, and positive feelings toward mental health. Two items were used to assess current clinical mental health practices: ‘In the past 12 months, have you used medication for a mental health problem’ and ‘In the past 12 months, have you received support (i.e. advice, care) for your mental or emotional health from a mental health professional (i.e. counselor, therapist, psychiatrist)?’ If participants answered yes to either of these questions, they were considered to have utilized clinical mental health services.

To assess the perceived need for mental health support, one yes or no item was asked, ‘In the past 12 months, did you think you needed help for emotional or mental health problems such as feeling sad, anxious, or nervous?’

To assess positive feelings toward using clinical mental health, participants were asked to rate the following two statements on a Likert scale (1 = strongly disagree, 5 = strongly agree): ‘I feel positive about using clinical mental health services (such as therapy and medication)?’ and ‘I think using clinical mental health services can be helpful for me.’ These items were averaged to measure how positively students felt toward using clinical mental health and dichotomized into high and low scores of positive views toward use.

### Independent variables

Scale items are available upon request to the corresponding author.

#### Relatedness toward using clinical mental health services

Relatedness was measured using two constructs: perceived stigma and proximity to mental health. The 12-item stigma subscale of the Barriers to Access to Care Evaluation scale (BACE) was used to assess perceived stigma (Clement *et al*., 2012) (Cronbach's *α* = 0.89). In order to assess proximity, the Reported and Intended Behaviour Scale (RIBS) (Evans-Lacko *et al*., 2011) was adapted. Current behavior was assessed using four yes/no questions. To measure whether one spoke to others about mental health, ‘How many people have talked to you about the importance of mental health?’ was asked. The number of people spoken to regarding the importance of mental health was totaled and averaged with the sum of the four yes/no responses to develop a (0–4) scale of mental health proximity. Four items from RIBS measured on a 1–5 Likert scale were used to measure intended conduct toward people with mental health problems. Answers were summed to create a final score (1–20).

#### Autonomy toward using clinical mental health services

Autonomy was measured using the BACE subscale for instrumental and attitudinal barriers. Participants rated statements about barriers to getting mental health services on a Likert scale, (1) not a barrier to (4) a lot (major barrier). The final result was dichotomized into (0 = )low/neutral or (1 = )high barriers. Four new instrumental barriers related to technology were added to the scale. The revised instrument had a Cronbach's *α* = 0.85.

#### Competency in using clinical mental health services

Six items were used from The Mental Health Knowledge Schedule (Evans-Lacko *et al*., 2010). Six questions were omitted from usage because they encompassed Western-defined mental disorder symptomology and were not culturally relevant. Questions were rated 0–4 on a scale of strongly agree to strongly disagree. A seventh item, ‘people with mental health problems typically seek help from a mental health professional’, was added to this scale. The scores were summed to create a 0–28 score, with higher values indicating stronger mental health knowledge.

#### Current nonclinical mental health practices

Measurements of nonclinical approaches to mental health were adapted from the Healthcare for Communities Study and Eisenberg *et al*.'s (2011) research examining mental health service utilization among college students in the United States.

To assess nonclinical mental health support practices, the following questions were asked: In the last year, did you receive mental or emotional health support from (1) friends, (2) family, (3) spouse, (4) religious leader, (5) teacher or coach, (6) other person, (7) social media/technology? Responses were summed, and a variable of 0–8 was created, indicating the number of support sources participants used for nonclinical mental health support.

The Brief COPE survey (Carver, 1997) measured students' existing coping strategies. Brief COPE items can be categorized as adaptive coping methods: acceptance, active, altruistic, emotional, informational, planning, positive reframing, and self-care. Responses were categorized into counts (0–8) of adaptive mechanisms used.

A single-item question from the PHQ-9, ‘Have you ever had thoughts that you would be better off dead or thoughts of hurting yourself in some way?’, measured lifetime suicidal ideation.

### Covariates

#### Perceived stress

The Perceived Stress Scale (PSS-4), established by Cohen *et al*. (1983), was used to assess stress (Cronbach's *α* = 0.70 in current sample). The final score is summed (0–16).

#### Depression

The PHQ-2 was used to assess depression symptoms (Ganguly *et al*., 2013). Participants rated feeling depressive symptoms on a scale from 0 (never) to 3 (almost daily). The optimal cut-point for likely having major depressive disorder was 3; thus, depressive symptomatology was divided into high (3+) and low depressive scores in this study (0–2 points).

#### Demographics

Gender was assessed as male, or female and gender minority. Age was a constant variable. Socioeconomic level growing up was measured as low (never, rarely, or occasionally) or high (most of the time or always) in terms of how often their family had enough money to make ends meet. Sexual orientation was categorized as straight/heterosexual or gay, lesbian, bisexual, asexual, uncertain, or self-describe. Relationship status was categorical: single, dating, or married; other: divorced, separated, widowed, or self-described. Degree type consisted of Bachelors, Master's, or Doctorate degree. The length of time in university was determined by their year of study (1–4+). Self-perception of religiosity was measured on a 0–10 scale.

### Data handling

A validity question was integrated into the survey to ensure participants accurately read questions. All responses from participants who answered the validity question incorrectly were removed from data analysis. Complete case analysis was used for all results.

### Statistical analysis

Participants' demographic characteristics, including age, gender, sexual orientation, childhood socioeconomic background, relationship status, year of schooling, degree of study, and university being attended, were reported using descriptive statistics.

*A priori α* level was set at 0.05. *R*^2^ was reported for the adjusted logistic regression model to explain the total variance. Analyses were conducted using the statistical package SPSS (SPSS 25, 2017). Pearson's correlations and collinearity diagnostics were used to assess possible multicollinearity between independent variables for each model; all value inflation factors were lower than 2, indicating no collinearity. Logistic regression was used to examine the bivariate relationship between independent variables using clinical mental health services and positive views of clinical mental health; odds ratios and *p* values with confidence intervals are reported. Based on the *p* values of the unadjusted logistic regression, only variables with *p* values of 0.20 or less were included in the final regression model (Hosmer *et al*., 1989). Adjusted odds ratios were reported for the final model, along with model fit statistics. Reliability analysis was also included, using Cronbach's *α* to report on the reliability of scales used in the study. Two-tailed significance was reported for all analyses.

## Results

Table 1 shows participant demographics for the total validated, a complete case sample size (*n* = 350). The overall mean age of the sample was 22.8 years (s.d. 2.17). In total, 57.1% of this sample identified as male, 41.7% as female, and 1.1% of the sample as a gender minority. Most of the sample identified as heterosexual or straight (94.2%), and 73.4% of the sample identified as their families having enough money to make ends meet all or most of the time while growing up. Much of the sample (76.3%) identified as single regarding relationship status. There was near equal distribution of first, second, third, and fourth-year or higher student respondents; 83.4% of the sample were pursuing a bachelor's degree. The sample represented 27 universities across Bangladesh, with the majority (62.8%) from Jahangirnagar University. Students identified as moderately religious (*M* = 6.33, s.d. = 2.00). Nearly half the sample (49.4%) reported feeling like they struggled with their mental health in the past year, and overall, students were moderately stressed (*M* = 8.46, s.d. = 3.41). In total, 43.7% of the sample had high depressive symptoms, and 28.3% of the sample had lifetime suicidal ideation. Students had moderate levels of wellness (*M* = 26.47, s.d. = 10.08).

**Table 1.** Participant demographics (*n* = 350)

	%/*M* (s.d.)
Age (18–41)	22.80 (2.17)
Gender	
Male	57.1%
Female and gender minority	42.9%
Sexual orientation	
Heterosexual/straight	94.2%
Sexual orientation minority (LGBTQA+)	5.8%
Childhood SES^a^	
Low	26.6%
High	73.4%
Relationship status	
Single	76.3%
Partnered (relationship, married)	22.9%
Other	0.9%
Year/semester in school	
1st–3rd/first year	20.4%
4th–6th/second year	18.7%
7th–9th/third year	19.8%
10th–12th/fourth year	20.4%
12th+/fourth year+	20.7%
Degree of study	
Bachelors (BS, BA)	83.4%
Masters (MPH, MBA)	16.6%
University	
Jahangirnagar University	62.8%
Bangladesh University of Business and Technology	6.1%
East-West University	4.4%
University of Dhaka	4.1%
University of Chittagong	3.2%
Other (*N* < 10 per school)	19.5%
Religiosity (1–10)	6.33 (2.00)
Perceived stress (0–16)	8.46 (3.41)
High depressive symptoms (>3)	43.7%
Suicidal ideation (lifetime)^b^	28.3%
COVID-19 impact on mental health	
Made it worse	41.0%
Stayed same	44.5%
Made it better	14.6%

*M*, mean; s.d., standard deviation.

a Item asked, how often did your family have enough money to make ends meet growing up, low = never, rarely, sometimes; high = most of the time, always.

b 315 respondents answered this item.

The majority of the sample (70.8%) either felt they needed support for their mental health (49.4%) or had high depressive scores (43.7%) or stress scores (28.0%), or had past suicidal ideation (27.9%). Nearly a quarter of the sample had problems with their mental health, but not perceive they needed help, for example, 76 of the participants (24% of the sample that responded to all pertinent items, *n* = 315) reported they did not need support for their mental health, while simultaneously reporting high depressive symptoms, stress, or past ideation. For this reason, the regression analysis is conducted on the full sample data, rather than a subsample of only those who reported needing mental health support.

As shown in Table 2, nearly half the population (49.4%) felt they needed help with their mental health. Respondents sought nonclinical support from an average of two sources. On average, participants engaged in five different types of adaptive coping: self-care was used most commonly (80.1%), and two types of maladaptive coping, distraction used most commonly (77.6%). Nearly all participants engaged in at least one mindful activity (96.5%) including identifying and prioritizing values, identifying and trying to change negative thoughts, deep breathing, de-stressing meditation, staying in the moment, focusing on senses, and activities to promote self-esteem.

**Table 2.** Use of mental health practices and services (*n* = 350)

	%/*M* (s.d.)
Perceived need for mental health support	49.4%
Nonclinical mental health	
Number of sources received nonclinical support from (0–7)^a^	2.38 (1.62)
Adaptive coping types (0–8)	4.78 (2.28)
Maladaptive coping types (0–5)	1.89 (1.12)
Participation in mindfulness activities	96.9%
Identifying and prioritizing values	79.7%
Identifying and trying to change negative thoughts	85.4%
Deep breathing, de-stressing, meditation, staying in the moment, focusing on senses	57.4%
Activities to promote self-esteem, such as gratitude journaling	37.4%
Clinical mental health	
Received clinical support from a therapist, psychiatrist, or mental health medication (past year)	7.1%
Feel positive about clinical mental health	54.3%
Stigma regarding using clinical mental health services (0–3)	0.67 (0.63)
Proximity to mental health (know someone with a mental health problem, talk to people about mental health) (0–4)	1.78 (1.12)
Willingness to interact with someone with a mental health problem (1–20)	12.69 (3.99)
High instrumental and attitudinal barriers to care	10.9%
Mental health knowledge (0–28)	16.0 (4.22)

*M*, mean; s.d., standard deviation, higher score equal greater amounts.

a Count of where sought nonclinical support from: friends, family, partner, faith leader, counselor, other person, or technology.

The majority of participants (54.3%) felt positive toward using clinical practices. Only 7.1% received clinical support from a therapist psychiatrist or used mental health medication in the past year.

When examining the construct of relatedness, participants reported low levels of perceived stigma (*M* = 0.68, s.d. = 0.65). The majority of the sample (78.3%) talked to at least one other person about the importance of mental health. On average, participants knew and spoke to approximately two people regarding the importance of mental health and were moderately willing (*M* = 12.69, s.d. = 3.99) to interact with someone who has a mental health problem.

When examining the construct of autonomy, only 10.9% of the sample perceived high levels of instrumental and attitudinal barriers to seeking mental health care. Regarding competency, respondents had moderate knowledge regarding mental health (*M* = 16.00, s.d. = 4.22), indicating they know how to combat mental health problems and correctly identify methods of seeking care and prognoses of mental health treatment.

### Regression results

Table 3 highlights the logistic regression results for factors predicting positive views regarding clinical health services. In the unadjusted regression, constructs of relatedness and competency were associated with greater positive views of clinical mental health services. Knowing people who have struggled with mental health problems or talking to others about the importance of mental health was associated with having positive views of clinical mental health services. However, in the adjusted model, only competency remained statistically significant (aOR = 1.10, *p* = 0.001). Having higher mental health knowledge – knowing how to help someone with a mental health problem and having accurate information regarding the efficacy of therapy, medication, and utilization of mental health services – was a significant predictor of having positive views regarding clinical health care. The *R*^2^ for the final adjusted model was 0.078 (*p* = 0.001).

**Table 3.** Logistic regression associating self-determination constructs-relatedness, autonomy, and competency with positive *v*. negative or neutral views of clinical mental health services, *N* = 350

	Unadjusted associations		Adjusted model	
	OR (95% CI)	*p*	aOR (95% CI)	*p*
Relatedness				
Stigma regarding clinical mental health services (0–3)	0.75 (0.54–1.04)	0.087	0.87 (0.57–1.33)	0.506
Proximity to mental health (0–4)	1.24 (1.02–1.50)	0.030	1.14 (0.93–1.39)	0.221
Willingness to interact with someone with mental health problem (1–20)	0.98 (0.93–1.04)	0.514		
Autonomy				
Instrumental and attitudinal barriers to care (high *v*. low)	0.51 (0.26–1.02)	0.055	0.71 (0.30–1.68)	0.440
Competency				
Mental health knowledge (0–28)	1.11 (1.05–1.17)	<0.001	1.10 (1.04–1.16)	0.001
Control variables				
Perceived stress (0–16)	0.98 (0.92–1.04)	0.519		
Depressive symptoms (low/high)	0.75 (0.49–1.15)	0.191	0.84 (0.53–1.32)	0.448
Religiosity (0–10)	1.02 (0.92–1.14)	0.714		
Socioeconomic status growing up (low to high)^a^	0.91 (0.57–1.47)	0.713		
Gender (female and gender minority *v*. male)	0.94 (0.61–1.43)	0.757		
Nagelkerke *R*^2^	−		0.078	
*p*	−		0.001	

*N* , sample size; aOR, adjusted odds ratio; 95% CI, 95% confidence interval, higher score equal greater amounts.

a Item asked, how often did your family have enough money to make ends meet growing up, low = never, rarely, sometimes; high = most of the time, always.

In the second logistic regression model, predictors of whether participants self-identify as needing mental health support are shown in Table 4. In step 2 of the adjusted model, positive views of clinical mental health services are added as a predictor, but there were no changes in significant findings between step 1 and step 2. Relatedness predictors of perceived stigma and proximity to mental health are significant in all models. In both adjusted models, those who perceived more stigma surrounding mental health (aOR 1.70, *p* = 0.023) and knowing or talking to more people with or about mental health (aOR = 1.31, *p* = 0.009) had increased odds of self-identifying as needing support for mental health. Females and gender minorities were 78% more likely than males to think they need support for their mental health (*p* = 0.013). Those with higher scores on perceived stress had greater likelihood of perceiving the need for mental health support (aOR = 1.14, *p* = 0.001). The *R*^2^ for the final adjusted model is 0.163 (*p* < 0.001).

**Table 4.** Logistic regression associating self-determination constructs-relatedness, autonomy, and competency with perceived need for mental health help (*N* = 350)

	Unadjusted associations		Step 1		Step 2	
	OR (95% CI)	*p*	aOR (95% CI)	*p*	aOR (95% CI)	*p*
Relatedness						
Stigma regarding clinical mental health services (0–3)	1.94 (1.36–2.76)	<0.001	1.69 (1.07–2.65)	0.024	1.70 (1.08–2.69)	0.022
Proximity to mental health (0–4)	1.29 (1.06–1.56)	0.010	1.34 (1.09–1.64)	0.005	1.31 (1.07–1.61)	0.009
Willingness to interact with someone with mental health problem (1–20)	0.99 (0.94–1.05)	0.838				
Autonomy						
Instrumental and attitudinal barriers to care (high *v*. low)	1.88 (0.94–3.76)	0.076	1.03 (0.42–2.49)	0.953	1.07 (0.44–2.61)	0.878
Competency						
Mental health knowledge (0–28)	1.02 (0.97–1.08)	0.335				
Control variables						
Perceived stress (0–16)	1.16 (1.09–1.24)	<0.001	1.13 (1.06–1.22)	0.001	1.14 (1.06–1.22)	0.001
Depressive symptoms (low/high)	2.06 (1.34–3.16)	0.001				
Religiosity (0–10)	0.95 (0.85–1.06)	0.350				
Socioeconomic status growing up (low to high)^a^	0.84 (0.52–1.35)	0.463				
Gender (female and gender minority *v*. male)	1.83 (1.19–2.81)	0.006	1.77 (1.12–2.79)	0.015	1.78 (1.13–2.82)	0.013
Positive *v*. negative or neutral views of clinical mental health services	1.32 (0.87–2.02)	0.192	–		1.41 (0.90–2.23)	0.138
Nagelkerke *R*^2^			0.153		0.160	
*p*			<0.001		<0.001	

*N*, sample size; aOR, adjusted odds ratio; 95% CI, 95% confidence interval, higher score equal greater amounts.

Depression was not included in the adjusted model, to address collinearity with perceived stress (*r* = 0.45), there were no appreciable changes when depression replaced stress in the adjusted model, of the significant findings: stigma aOR = 1.83 (*p* = 0.008), proximity aOR = 1.33 (*p* = 0.005), depression aOR = 1.92 (*p* = 0.005), gender aOR = 2.00 (*p* = 0.003).

a Item asked, how often did your family have enough money to make ends meet growing up, low = never, rarely, sometimes; high = most of the time, always.

The unadjusted and adjusted logistic regression analyses for the outcome of clinical mental health use are presented in Table 5. The predictors of positive views toward mental health services and perceived need for mental health support were added in a step-wise fashion. In step 1 of the model, those who are more proximal to mental health, for example, knowing more people who may deal with mental health problems, have a higher adjusted odds ratio of receiving clinical health care (aOR = 1.47, *p* = 0.043). However, this finding did not remain significant when the construct of positive views toward clinical mental health services is added as a covariate, and was found to be significantly associated (aOR = 2.87, *p* = 0.033) to the outcome in step 2. Once the construct of perceived need is added to the model in step 3, we found that this was the largest driver of clinical service use, as those who perceive help were 400% more likely to receive clinical mental health care. For all steps of the model, higher religiosity levels indicated lower odds of receiving clinical health care (aOR = 0.80, *p* = 0.041). The *R*^2^ for the final adjusted model was 0.181 (*p* < 0.001).

**Table 5.** Logistic regression associating self-determination constructs-relatedness, autonomy, and competency with the use of clinical services, *N* = 350

	Unadjusted associations		Step 1		Step 2		Step 3	
	OR (95% CI)	*p*	aOR (95% CI)	*p*	aOR (95% CI)	*p*	aOR (95% CI)	*p*
Relatedness								
Stigma regarding clinical mental health services (0–3)	1.57 (0.87–2.81)	0.133	1.46 (0.69–3.10)	0.319	1.37 (0.37–5.02)	0.640	1.21 (0.33–4.41)	0.778
Proximity to mental health (0–4)	1.48 (1.02–2.13)	0.037	1.47 (1.01–2.12)	0.043	1.39 (0.96–2.02)	0.085	1.28 (0.86–1.89)	0.223
Willingness to interact with someone with mental health problem (1–20)	1.01 (0.91–1.12)	0.882						
Autonomy								
Instrumental and attitudinal barriers to care (high *v*. low)	2.21 (0.78–6.28)	0.136	1.43 (0.37–5.52)	0.603	2.14 (0.57–8.12)	0.263	2.01 (0.53–7.61)	0.305
Competency								
Mental health knowledge (0–28)	1.06 (0.96–1.17)	0.267						
Control variables								
Perceived stress (0–16)	1.02 (0.90–1.14)	0.801						
Depressive symptoms (low/high)	1.21 (0.53–2.72)	0.654						
Religiosity (0–10)	0.80 (0.66–0.97)	0.024	0.79 (0.65–0.97)	0.025	0.81 (0.66–0.99)	0.036	0.80 (0.65–0.99)	0.041
Socioeconomic status growing up (high *v*. low)^a^	1.16 (0.45–2.99)	0.763						
Gender (female and gender minority *v*. male)	1.49 (0.66–3.36)	0.340						
Positive *v*. negative or neutral views of clinical mental health services	2.85 (1.11–7.33)	0.029	–		2.87 (1.09–7.59)	0.033	2.63 (0.98–7.03)	0.054
Perceived need (need *v*. no need)	5.98 (2.01–17.79)	0.001	–		–		4.99 (1.64–15.21)	0.005
Nagelkerke *R*^2^	−		0.083		0.112		0.181	
*p*	−		0.019		0.006		<0.001	

*N*, sample size; aOR, adjusted odds ratio; 95% CI, 95% confidence interval, higher score equal greater amounts.

a Item asked, how often did your family have enough money to make ends meet growing up, low = never, rarely, sometimes; high = most of the time, always.

## Discussion

The prevalence of mental health problems in this sample is similar to other studies in Bangladeshi university populations at a similar period. Depression levels in this sample are high (44%) and comparable to other Bangladeshi student populations (Islam *et al*., 2020; Khan *et al*., 2020; Koly *et al*., 2021). Though only 7.1% of our sample used clinical services, the vast majority (87.1%) sought nonclinical or informal help. Half the sample (50.8%) preferred getting help from their friends or family as opposed to seeking clinical help. This finding is similar to Bilican (2013) who found that Turkish college students preferred social support over psychotherapy. Over half the sample engaged in about five out of eight adaptive coping strategies; this finding supports other research during the COVID-19 pandemic, where students used a variety of stress-management techniques (Ahmed *et al*., 2021).

As described by SDT, we hypothesized that autonomy, relatedness, and competency predict perceived need for mental health support, use of nonclinical practices and clinical services, and positive beliefs toward using clinical services; our findings partially confirmed these hypotheses. Perceived need for mental health support and use of clinical services were associated with relatedness characteristics factors, whereas competency was linked to positive views toward clinical mental health care utilization. Higher competency, particularly mental health literacy, can increase positive views of clinical mental health, and higher relatedness can increase ones' perceived need for services. Positive attitudes toward services increase the likelihood of usage, but perceived need is a stronger predictor of utilization. The results provide significant insight into mental health recognition and coping techniques used by Bangladeshi university students.

The findings suggest that positive views of mental health care are driven by competency. This is similar to findings from college populations in the United States, where mental health literacy was found to be a predictor of positive attitudes toward, and actual help-seeking behavior (Cheng *et al*., 2018). Relatedness was indicative of perceived need and use of mental health care. Relatedness factors can increase mental health competency, for example, people who know others with mental health issues are more likely to seek help (Vogel *et al*., 2007; Kearns *et al*., 2105), because they are more familiar with the topic. While one study in Bangladesh found stigma as a significant barrier to help-seeking (Koly *et al*., 2021), no studies have looked at this as a predictor of perceived need for care. Contrary to our expectations, we found that higher levels of stigma were associated with feeling like one needed mental health help. Perhaps because people who have considered care have also contemplated the alleged effects of the treatment, and thus feel stigmatized by the subject.

While not a main study aim, it was also expected that religiosity would be a protective factor for mental health, as found in other studies based in South Asian countries (Nadeem *et al*., 2017). Devine *et al*. (2019) found that having a strong religious identity in Bangladesh was associated with higher wellness. In the current sample, higher religiosity was correlated with higher overall mental wellness (*r* = 0.24, *p* < 0.001) and lower perceived stress (*r* = −0.13, *p* = 0.016); however, we found higher religiosity levels to be negatively associated with the use of mental health care. This may be due to the use of religiosity or spirituality as a coping mechanism rather than clinical care (Nuri *et al*., 2018).

Examining this from a theoretical standpoint, these findings suggest that seeking mental health care stems from internalized motivation. Internalized motivation encompasses doing something because it is useful and aligns with ones' values. Given 70.8% of the sample either felt they could use mental health support or were actually dealing with mental health problems (stress, depression, past suicidal ideation), it is important to look at the entire sample to examine what influences positive views, perceived need, and use of clinical services. We see that knowledge of mental health drives ones' positive views of mental health services, and that higher levels of stress and being close to people with mental health problems is associated with perceived necessity of mental health support, which in turn is the main driver of actually using clinical health services. Future studies should examine internalized motivation as it relates to seeking mental health care. The outcomes of this study were chosen due to how SDT places perceived usefulness (conceptualized as perceived need) and value (positive views of clinical services) driven motivations on a spectrum of motivation. Future directions should establish a time order of motivation and examine pathways from these outcomes of positive feelings toward mental health services, perceived necessity, and engagement in mental health care.

### Limitations

The convenience sampling, cross-sectional survey, and self-report nature of the data collection only allow for associations between variables to be examined; it does not allow for broad generalizability time order to be established, and opens the data up for social desirability bias. Given the title of the study, people who were more versed in mental health may have been self-selected, which would cause low levels of perceived stigma and high levels of mental health problems.

### Practical implications

Given the use of a culturally adaptive framework and extensive community engagement in the survey development and data collection phases, this study provides culturally competent evidence that emphasizes how crucial it is for Bangladeshi students to identify their mental health care needs. Universities can promote educational campaigns across campus, educating students about the warning signs of deteriorating mental health (Bhuiyan *et al*., 2020); this is particularly important as students have an overall low level of mental health literacy (Arafat *et al*., 2019). Given that students are open to using adaptive coping strategies, providing students with opportunities to engage in these strategies would be beneficial. As relatedness was associated with acknowledgement of need and actual use of clinical services, and nearly half the sample reports having difficulty with mental health, speaking transparently about the topic could be greatly influential. In short, universities should help students to (a) view mental health positively by increasing their knowledge on the topic, including its prevalence of mental health issues among young adults, (b) provide safe spaces for students to have an open dialogues about mental health in order to help students recognize that they need to seek help as early as possible for better outcomes, and finally (c) provide resources for students to receive care should they seek it. Past research supports these methods, as interventions that provide a space to allow someone who experienced mental health problems to share their stories with college students, and providing education that designs mental illness and stigma are shown to decrease stigma related to mental health (Kosyluck *et al*., 2016). Interviews with students corroborate these findings, and believe that educating students, providing them with resources, and being compassionate and understanding will help decrease barriers to receiving mental health care (Vidourek and Burbage, 2019).
